# Divergent colonization traits, convergent benefits: different species of arbuscular mycorrhizal fungi alleviate *Meloidogyne incognita* damage in tomato

**DOI:** 10.1007/s00572-024-01139-7

**Published:** 2024-03-05

**Authors:** Milena Caccia, Nicolás Marro, Václav Novák, Juan Antonio López Ráez, Pablo Castillo, Martina Janoušková

**Affiliations:** 1 Institute of Botany, Czech Academy of Sciences, Zámek 1, Průhonice, 252 43 Czech Republic; 2 Instituto Multidisciplinario de Biología Vegetal (IMBIV), CONICET, FCEFyN, Universidad Nacional de Córdoba, CC, 495, 5000, Córdoba, Argentina; 3 Department of Soil and Plant Microbiology, Estación Experimental del Zaidín (EEZ-CSIC), C/ Profesor Albareda 1, 18008 Granada, Spain; 4 Institute for Sustainable Agriculture (IAS), Spanish National Research Council (CSIC), Campus de Excelencia Internacional Agroalimentario, ceiA3, Av. Menéndez Pidal s/n, 14004 Córdoba, Spain

**Keywords:** Root knot nematodes, Arbuscular mycorrhizal fungi, Biological control, Plant nutrition

## Abstract

Arbuscular mycorrhizal fungi (AMF) can increase plant tolerance and/or resistance to pests such as the root-knot nematode *Meloidogyne incognita*. However, the ameliorative effects may depend on AMF species. The aim of this work was therefore to evaluate whether four AMF species differentially affect plant performance in response to *M. incognita* infection. Tomato plants grown in greenhouse conditions were inoculated with four different AMF isolates (*Claroideoglomus claroideum*,* Funneliformis mosseae*,* Gigaspora margarita*, and* Rhizophagus intraradices*) and infected with 100 second stage juveniles of *M. incognita* at two different times: simultaneously or 2 weeks after the inoculation with AMF. After 60 days, the number of galls, egg masses, and reproduction factor of the nematodes were assessed along with plant biomass, phosphorus (P), and nitrogen concentrations in roots and shoots and root colonization by AMF. Only the simultaneous nematode inoculation without AMF caused a large reduction in plant shoot biomass, while all AMF species were able to ameliorate this effect and improve plant P uptake. The AMF isolates responded differently to the interaction with nematodes, either increasing the frequency of vesicles (*C. claroideum*) or reducing the number of arbuscules (*F. mosseae* and *Gi. margarita*). AMF inoculation did not decrease galls; however, it reduced the number of egg masses per gall in nematode simultaneous inoculation, except for *C. claroideum*. This work shows the importance of biotic stress alleviation associated with an improvement in P uptake and mediated by four different AMF species, irrespective of their fungal root colonization levels and specific interactions with the parasite.

**Supplementary information:**

The online version contains supplementary material available at 10.1007/s00572-024-01139-7.

## Introduction

Food production is a major concern for the growing human population. The intensive use of soil to increase its productivity has led to its degradation, affecting ecosystems, environment, and human health. Consequently, there is a need to seek sustainable solutions and new approaches in agriculture to restore the quality of ecosystem services, in which soil microbiota are crucial. Among soil microorganisms, arbuscular mycorrhizal fungi (AMF) establish mutualistic relationships by colonizing the roots of most plant species (Turrini et al. 2018). In addition to nutritional benefits, AMF can increase plant tolerance and/or resistance to abiotic and biotic stresses such as pathogenic fungi, bacteria, viruses, parasitic plants, and nematodes (Diagne et al. 2020). Mycorrhizal protection against biotic stress is becoming widely acknowledged in the context of plant production and is subject to the influences of various biotic and abiotic factors (Dowarah et al. 2022). In this sense, different AMF species differ in their interactions with host plants, yet the mechanisms behind these interaction differences require further investigation (Marro et al. 2022).

Plant-parasitic nematodes (PPN) establish an intimate relationship with their host in which they can reprogram plant cells for their own benefit (Phani et al. 2021). There are more than 4100 species of PPN, and some of them have great impact on agriculture and horticulture worldwide, accounting for annual losses of £173 billion (Decraemer and Hunt 2006). Among PPN, *Meloidogyne* (Tylenchida - Phylum: Nematoda), commonly known as the root-knot nematode, is one of the most devastating genera (Jones et al. 2013). The second stage juvenile (J2) penetrates the roots through a stylet and migrates close to the vascular cylinder, where it selects several cells to induce the formation of a feeding site (Perry and Moens 2011) and become sedentary. The surrounding cortex cells divide and become hypertrophied and the pericycle cells proliferate resulting in the formation of a root swelling known as a gall. This alters the root’s uptake of nutrients and causes symptoms of wilting and yield reduction (Castagnone-Sereno et al. 2013). Inside the gall, the J2 moults to J3, J4 and finally adult female, acquiring a pear-shaped form. When mature, the female lays eggs in an egg mass consisting of a gelatinous matrix on the root surface. The first juvenile molts within the egg to the infective or J2 stage, which emerges from the egg into the soil and can seek new hosts (Subedi et al. 2020). So far, the main management strategy used against this pest is through chemical control, which has detrimental effects on soils and non-target species (Abd-Elgawad 2021). Resistant genes against *Meloidogyne* spp. have also been described for tomato (Mi genes) (e.g., Ammiraju et al. 2003) and some other horticultural species (see Escobar et al. 2015). However, resistant cultivars are not effective against certain breeds of *Meloidogyne* spp. and can lose their resistance at high soil temperatures (de Almeida et al. 2020). The use of AMF as a biological control agent against PPN has been documented in the scientific literature, as AMF can reduce PPN populations (e.g., Castillo et al. 2006; Herrera-Parra et al. 2021; Marro et al. 2014, 2018). Proposed mechanisms of PPN control include direct effects, such as competition for space and nutrients, and indirect (plant-mediated) effects (Schouteden et al. 2015). AMF could protect roots from nematode invasion, either through their intricate hyphal networks in the soil or through competition for space in the roots. However, this hypothesis has yet to be proven (Dowarah et al. 2022). With regards to plant-mediated responses to nematodes, mycorrhizal plants might be more capable of handling the stress caused by *Meloidogyne* spp. because of enhanced ability to recover from damage following root nematode infection (Banuelos et al. 2014). Moreover, AMF may induce either localized and systemic resistance mechanisms in plants (Vos et al. 2012b) by inducing a priming effect (Vos et al. 2013; Molinari and Leonetti 2019; Pozo and Azcón-Aguilar 2007) and can reduce root penetration by PPN through changes in root exudates (Vos et al. 2012a, b).

The biocontrol effect of AMF against PPN depends on several factors. One of them may be the AMF species involved, as different AMF isolates are expected to affect plant growth, nutrition, and stress responses differently (Mensah et al. 2015; Munkvold et al. 2004; Sikes et al. 2009). Most studies testing plant responses to AMF have used *Rhizophagus irregularis* and *Funneliformis mosseae* (Glomerales), as they are considered generalists and ubiquitous species; however, these species can be quite heterogeneous in their functionality (Berruti et al. 2016). It has been observed that either *Rhizophagus* spp. or *F. mosseae* have an advantage over other AMF species in root colonization on different plant hosts (e.g., Blažková et al. 2021; Carrara and Heller 2022; Jansa et al. 2008; Säle et al. 2021; Voříšková et al. 2019). A rapid colonization rate could provide them with greater ability to compete with PPN for space in the rhizosphere and/or the roots. Regarding nutritional effects, it has been demonstrated, for example, that plants inoculated with *F. mosseae* have highly efficient water uptake (Marulanda et al. 2003), while those with *Rhizophagus* spp. have enhanced P acquisition, and plants with *Claroideoglomus* spp. and *Gigaspora* spp. show increased magnesium and calcium uptake (Carrara and Heller 2022). Also, a recent study found that different genetic organization (dikaryotic versus homokaryotic) of strains within the same species (*Rhizophagus irregularis*) can differentially affect the response of plants to mycorrhizas (Terry et al. 2023). Such differences may contribute to different tolerance of the host plant to nematode attack.

Finally, AMF species induce different systemic responses in plants, which may lead to differences in resistance to nematodes. Immunity in plants is regulated by several phytohormones, mainly salicylic acid (SA), jasmonic acid (JA), and ethylene (ET). During AMF colonization of roots, plant defenses are activated and a cross-talk between SA and JA occurs. Mycorrhizal symbiosis primes the plant tissues for rapid effective activation of JA-dependent defenses upon attack, resulting in enhanced resistance (Pozo and Azcón-Aguilar 2007). This priming effect has been observed in tomato plants with a mixture of AMF, antagonistic fungi, and rhizobacteria against *M. incognita* by the up-regulation of various tested genes, such as PR- and ACO (Molinari and Leonetti 2019). Moreover, a study comparing the transcriptional response of tomato to *F. mosseae* and *R. irregularis* found that, although both species induced common oxylipin pathway genes related to JA biosynthesis, the overall transcriptional profiles by these two AMF were different (López-Ráez et al. 2010). Interestingly, the oxylipin pathway has been shown to be involved in plant nematode responses (Gao et al. 2008).

Although several studies have investigated the interactions among different plant species, nematodes, and AMF, they generally focus more on biocontrol than on nutrient acquisition. The novelty of this study lies in simultaneously comparing AMF species belonging to different families to explore whether they differentially affect plant nutrition and responses to nematode infection. We selected tomato (*Solanum lycopersicum* L.), a globally cultivated crop susceptible to several pests and pathogens including *M. incognita*, to evaluate whether four AMF species of contrasting functional characteristics differentially affect (a) plant stress responses to *M. incognita* infection and (b) development and reproduction of the nematode in relation with the plant response to AMF inoculation.

## Materials and methods

### Experimental design

The experimental design combined two factors in a full factorial manner. The first factor was inoculation with AMF, comprising five levels: uninoculated control (NM); and inoculation with one of four isolates: (1) *Rhizophagus intraradices* C. Walker and Schuessler (2010), isolate PH5 (RI), (2) C*laroideoglomus claroideum* C. Walker and Schuessler (2010), isolate BEG23 (CC), (3) *Gigaspora margarita* W. N. Becker & I. R. Hall (1976), isolate BEG34 (GM), and (4) *Funneliformis mosseae* C. Walker and Schuessler (2010), isolate BEG95 (FM). The second factor was infection with the nematode *Meloidogyne incognita* Kofoid and White (1919), with three levels: no nematodes added (NN), nematodes inoculated simultaneously with AMF at the establishment of the experiment (NS), and nematodes inoculated 14 days after the AMF inoculation (NP). Two different times of nematode application were included because the timing of *Meloidogyne* infection in relation to transplanting time and mycorrhiza establishment may play a role in the interaction (Talavera et al. 2001). Each treatment had 5 independent replicates (5 × 3 × 5 = 75 plants in total). The experiment was harvested 60 days after its establishment.

### Plant material and substrate

Tomato seeds (cv. Money maker) were surface sterilized in 10% NaOCl for 5 min, washed with distilled water, and sown in Petri dishes for germination at room temperature. Seedlings were grown in trays in a mixture of sterile sand and zeolite (1:1) for 4 weeks. The experiment was established by transplanting four-leafed seedlings individually into experimental pots (11 cm height × 13 cm diameter) containing 700 ml of the same substrate as used for pre-cultivation.

### Inoculation with AMF and nematodes

The AMF isolates are maintained at the Department of Mycorrhizal Symbiosis (Institute of Botany, Czech Academy of Sciences, Průhonice, Czech Republic) in an inert sand-zeolite mixture (1:1, v: v) with *Desmodium* sp. as host plant. The inoculum of each isolate consisted of homogenized substrate of approximately 6-month-old cultures with chopped roots, air-dried for 1 day. At the time of transplanting, each seedling was inoculated with 30 ml of the treatment-specific AMF inoculum mixed with 270 ml of sterile sand-zeolite (1:1, v: v) as a central substrate layer in the pot. For *Gi. margarita* only, 100 spores per pot, collected from sporulating cultures, were used as inoculum, as spores are the main infective propagules of *Gigaspora* spp. (Klironomos and Hart 2002). The control plants (NM) received the same amount of heat-sterilized substrate of AMF inoculum (autoclaved twice at 121 °C for 30 min, 24 h apart). To include similar soil microorganisms other than AMF into the NM treatments, 5 ml of a microbial filtrate was added to each pot: this was prepared by shaking 100 g of the nonsterile soil from a culture substrate with 1 L of deionized water for 30 min and filtering twice through filter paper (pore size 10 μm).

An isolate of *M. incognita* race 1 was obtained from roots of naturally infected *Vitis* rootstocks (Richter 110) at Bollullos par del Condado (Huelva province, Spain) (Gutiérrez-Gutiérrez et al. 2011). To establish and maintain *M. incognita*, the nematode isolate was raised on tomato plants starting from a single egg mass and subsequently reared in pots containing a mixture of sterile sand and zeolite (1:1) in which tomato plants (cv. Money maker) were grown under greenhouse conditions. After approximately 2 months, egg masses were extracted from the root galls and placed in Petri dishes containing sterile water. They were kept at room temperature (25 °C) until the eggs hatched (about 10 days). For inoculation, mobile J2 were collected with a pipette and counted under a stereomicroscope. Inoculation was performed with 100 J2 in 1 ml of distilled water by pipetting the larvae on the surface of the pot, next to the plant. This procedure was carried out in the same way for the NS and NP treatments, i.e., immediately after the establishment of the experiment or 14 days later, respectively.

### Growth conditions and harvest

Plants were grown under greenhouse conditions (June–August). They were watered daily and fertilized twice a week with Hewitt’s nutrient solution (Hewitt 1966) with phosphorus reduced to 25% of the standard concentration (H_2_NaPO_4_ 0.33 mM).

Sixty days after the inoculation of AMF treatments, the plants were harvested, and the roots gently washed. A sub-sample of the root system (approximately 0.5 g fresh weight) was stored in KOH for subsequent staining with trypan blue (Koske and Gemma 1989). The frequency of AMF structures in the stained roots (30 1.5-cm root segments per sample) was estimated by the grid line intersection method (Mc Gonigle et al. 1990), 100 intersecting lines per sample were scored at 100x magnification (Olympus Bx60). Hyphae, arbuscules, and vesicles, as the main intraradical structures of AMF, were scored separately, and the percentage of hyphal presence was taken as the total percentage of root colonization, as none of the scored lines intersected arbuscules or vesicles without also intersecting a hypha. The relative abundances of arbuscules (relA%) and vesicles (relV%) were calculated as their percentage frequencies within the colonized root, i.e., relA% = A / H × 100, where A and H are the frequencies of arbuscules and hyphae, respectively, as estimated by microscopy.

The remaining roots were observed under a stereomicroscope to count the number of galls and egg masses. Egg masses were removed and immersed in a 1% NaClO solution for 4 min to dissolve the gelatinous matrix (Hussey and Barker 1973), and the eggs were counted. The reproductive factor (RF) was calculated as follows: RF = final population / initial population, where final population is the number of eggs counted at the end of the experiment and initial population is the number of inoculated J2 (100). Roots and shoots were then dried at 60 °C to estimate their dry weight.

Subsamples of homogenized shoot and root biomass were ground using a Retsch MM200 mill (Retsch GmbH, Haan, Germany) to determine the P and N concentrations in the shoots of the experimental plants. N concentrations were determined using a Flash EA 2000 elemental analyzer coupled with a Delta V Advantage isotope ratio mass spectrometer (Thermo Fisher Scientific, Waltham, MA, USA). P concentrations were determined after mineralization (adding 4 ml of HNO_3_ and 1 ml of 30% H_2_O_2_) by the photometric method based on the reaction of phosphates with ammonium molybdate, using the reaction mixture with sulphuric acid, ascorbic acid, and tartarate antimonylo-potassium (Murphy and Riley 1962). The absorbance of the resulting blue color was measured with a UV-vis spectrophotometer UV-400 Unicam at 630 nm (Zbíral 1994). The P and N contents in shoots and roots were then calculated by multiplying the concentrations by the shoot or root dry weights. Shoot to root ratios of N and P were calculated from the elements’ concentrations.

### Data analysis

All collected parameters were subjected to two-way analysis of variance (ANOVA) with AMF and nematodes as the main effects including the interaction term. The Di Rienzo, Guzmán and Casanoves (DGC) test (*p* ≤ 0.05) was used to compare means *a posteriori* (Di Rienzo et al. 2002). The DGC test is based upon clustering and yields non-overlapping groups of homogenous means. When there was a significant interaction between the main factors, pairwise differences across all the treatments were considered, whereas when the interaction was not significant, differences were assessed by DGC only for significant main factors. Pearson correlation coefficients between root and shoot nutrient concentrations, dry weights, and nematode galls were performed, and their significance levels were estimated. Data were checked for normality and homogeneity of variance prior to statistical analysis. Model diagnostics were performed examining the model’s simulated quantile scaled residuals using the DHARMA package in R. The models were assessed for over-dispersion, zero-inflation, and expected distribution of the residuals (Hartig and Lohse 2020). When analyzing root colonization data, the NM control was excluded, and the data were square-root transformed to adjust for normality and homogeneity of variance. For analysis of the relV% parameter, the FM and GM inoculated treatments were excluded because these two isolates did not form vesicles. When analyzing the nematode parameters, the control without nematodes was excluded. The N shoot to root ratio was square-root transformed to adjust for normality and homogeneity of variances. A *posteriori* comparisons were performed on the transformed data. All these analyses were performed using the statistical software InfoStat/Professional (Infostat 2011) and its interface with the software R (R Core Team 2011).

## Results

### Root colonization

**Fig. 1:**
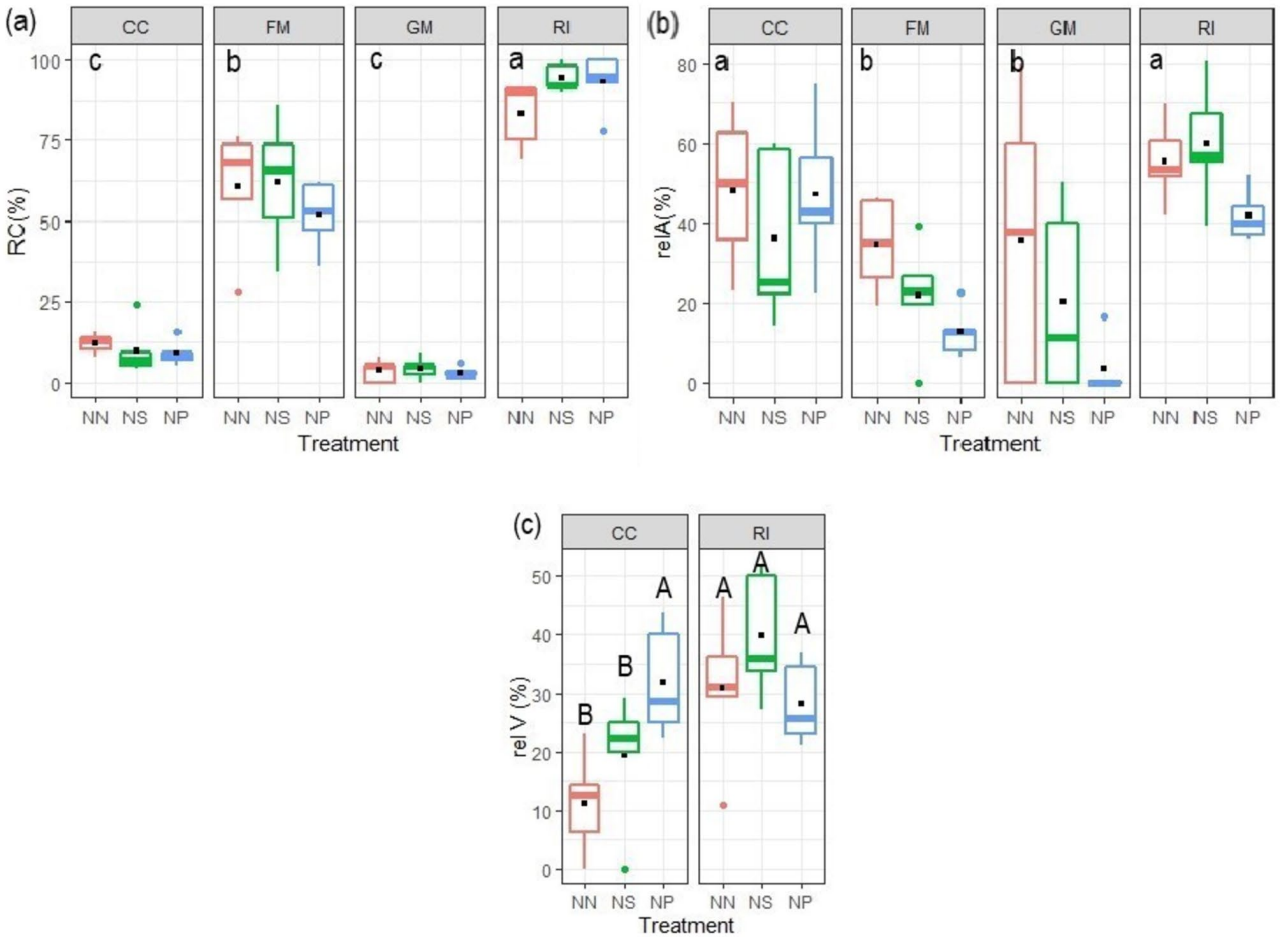
**a** Root colonization by four arbuscular mycorrhizal fungal (AMF) isolates, **b** relative abundance of arbuscules (relA%), and **c** of vesicles (relV%) of tomato plants subjected to different *M. incognita* treatments: no nematodes (NN), nematodes inoculated simultaneously with the AMF at planting (NS) or 2 weeks after AMF (NP). The AMF inoculations treatments were non-inoculated control (NM), inoculated with *C. claroideum* (CC), *F. mosseae* (FM), *Gi. margarita* (GM), or *R. intraradices* (RI). Different capital letters indicate significant differences across all treatments according to the DGC test (*p* < 0.05). Differences according to the DGC test (*p* < 0.05) for the AMF main factor effect are shown as lowercase letters at the upper left of each fungus panel when the interaction with nematode inoculation is not significant. In **a**, data were square root transformed to adjust for normality and homogeneity of variance. The box plots show the 25% and 75% quartiles with median, 1.5 times interquartile range (as whiskers) and outliers. Outliers were included in all analyses. Black squares indicate the mean values of each treatment (*n* = 5). Statistics are presented in Table 1

The four AMF isolates colonized roots with different rates (Table 1; Fig. 1), whereas no mycorrhization was observed in the non-inoculated plants of the NM treatment (data not shown). The highest root colonization was observed in RI plants with percentages ranging between 85 and 95%, whereas the lowest colonization rates were determined in GM (3–5%) and CC (12–21%) (Fig. 1a). No differences in root colonization were observed between the nematode treatments, nor was the interaction between the two factors significant (Table 1). The relA% was significantly influenced by the inoculation treatment (Table 1, main factor effect of AMF), lower in GM and FM than in RI and CC (Fig. 1b). Also, both nematode treatments (NS and NP) significantly reduced relA% overall (Table 1 main factor effect of Nematode). The effects of both main factors resulted in FM and GM having the lowest relA% when nematodes were applied. The relV% had a significant interaction among nematode and AMF treatments for the two fungi that formed vesicles: while nematode infection had no effect on relV% of RI, posterior nematode application increased relV% of CC (Fig. 1c, Table 1). FM and GM did not form vesicles.

**Table 1:** Effects of fungal isolate (AMF), nematode application (nematode), and the interaction of both factors on total root colonization by the arbuscular mycorrhizal fungus (RC), relative frequency of arbuscules (relA%), and vesicles (relV%)

	**AMF**			**Nematode**			**AMF x nematode**		
	d.f.	F	*p*	d.f.	F	*p*	d.f.	F	*p*
RC	3	244.27	**< 0.0001**	2	0.64	0.53	6	0.95	0.46
relA%	3	11.77	**< 0.0001**	2	4.58	**0.01**	6	1.14	0.35
relV %	1	138.47	**< 0.03**	2	3.12	0.1	2	3.02	**0.02**

*F*-values and significances are given according to ANOVA (Type III Sums of Squares)

Significant effects are highlighted in bold

### Plant growth and nutrition

#### Dry weights

**Fig. 2:**
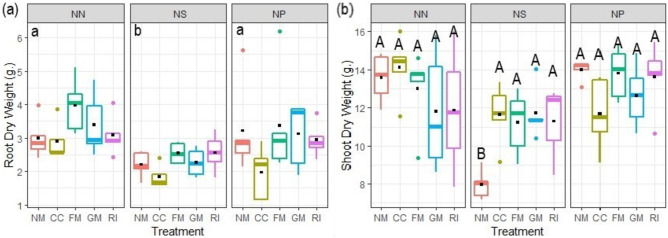
Root dry weight (**a**) and shoot dry weight (**b**) of tomato plants inoculated with four arbuscular mycorrhizal fungal (AMF) isolates and subjected to different *M. incognita* treatments: no nematodes (NN), nematodes inoculated simultaneously with the AMF at planting (NS) or 2 weeks after AMF (NP). The AMF inoculations treatments were non-inoculated control (NM), inoculated with *C. claroideum* (CC), *F. mosseae* (FM), *Gi. margarita* (GM), or *R. intraradices* (RI). Different capital letters indicate significant differences across all treatments according to the DGC test (*p* < 0.05). Differences according to the DGC test (*p* < 0.05) for the nematode main factor effect are shown as lowercase letters at the upper left of each fungus panel when the interaction with nematode inoculation is not significant. The box plot shows the 25% and 75% quartiles with median, 1.5 times interquartile range (as whiskers) and outliers. Outliers were included in all analyses. Black squares indicate the mean values of each treatment (*n* = 5). Statistics are presented in Table 2

Root dry weight was significantly affected by nematode infection and AMF inoculation, while the interaction of both factors was non-significant (Table 2). Simultaneous infection with nematodes decreased root dry weight as compared to the other two nematode treatments (Fig. 2a), the main effect of AMF inoculation consisted in significantly lower root dry weight of CC-inoculated plants as compared to plants in the other inoculation treatments. Shoot dry weight was not affected by AMF inoculation alone, but by the interaction of the two experimental factors (Table 2), inoculation with all AMF isolates alleviated the detrimental effect of nematodes on non-mycorrhizal plants after simultaneous nematode application (Fig. 2b).

**Table 2:** Effects of inoculation with arbuscular mycorrhizal fungi (AMF), nematode application (nematode), and the interaction of both factors on plant growth parameters, the concentrations and contents of phosphorus (P) and nitrogen (N)

	**AMF**		**Nematode**		**AMF x nematode**	
d.f.	4		2		8	
	F	*p*	F	*p*	F	*p*
Root dry weight	3.02	**0.025**	8.8	**0.0005**	0.55	0.82
Shoot dry weight	0.48	0.75	12.37	**< 0.0001**	3.0	**0.006**
Root N concentration	0.68	0.61	41.48	**< 0.0001**	2.47	**0.02**
Root P concentration	1.36	0.26	28.90	**< 0.0001**	2.88	**0.009**
Shoot N concentration	1.98	0.11	5.36	**0.007**	3.65	**0.002**
Shoot P concentration	0.43	0.79	5.27	**0.008**	1.29	0.27
Shoot P content	0.69	0.60	18.0	**< 0.0001**	2.83	**0.01**
Root P content	2.68	**0.04**	0.14	0.87	0.66	0.72
Shoot N content	1.70	0.16	9.14	**0.0004**	1.31	0.26
Root N content	1.76	0.15	0.94	0.40	0.87	0.55
N to P ratio shoots	2.11	0.09	9.73	**0.0002**	4.20	**0.0005**
N shoot to root ratio	1.41	0.24	23.86	**< 0.0001**	3.13	**0.005**
P shoot to root ratio	0.25	0.91	24.49	**< 0.0001**	2.95	**0.008**

*F*-values and significances are given according to ANOVA (Type III Sums of Squares)

Significant effects are highlighted in bold

#### Phosphorus and nitrogen

**Fig. 3:**
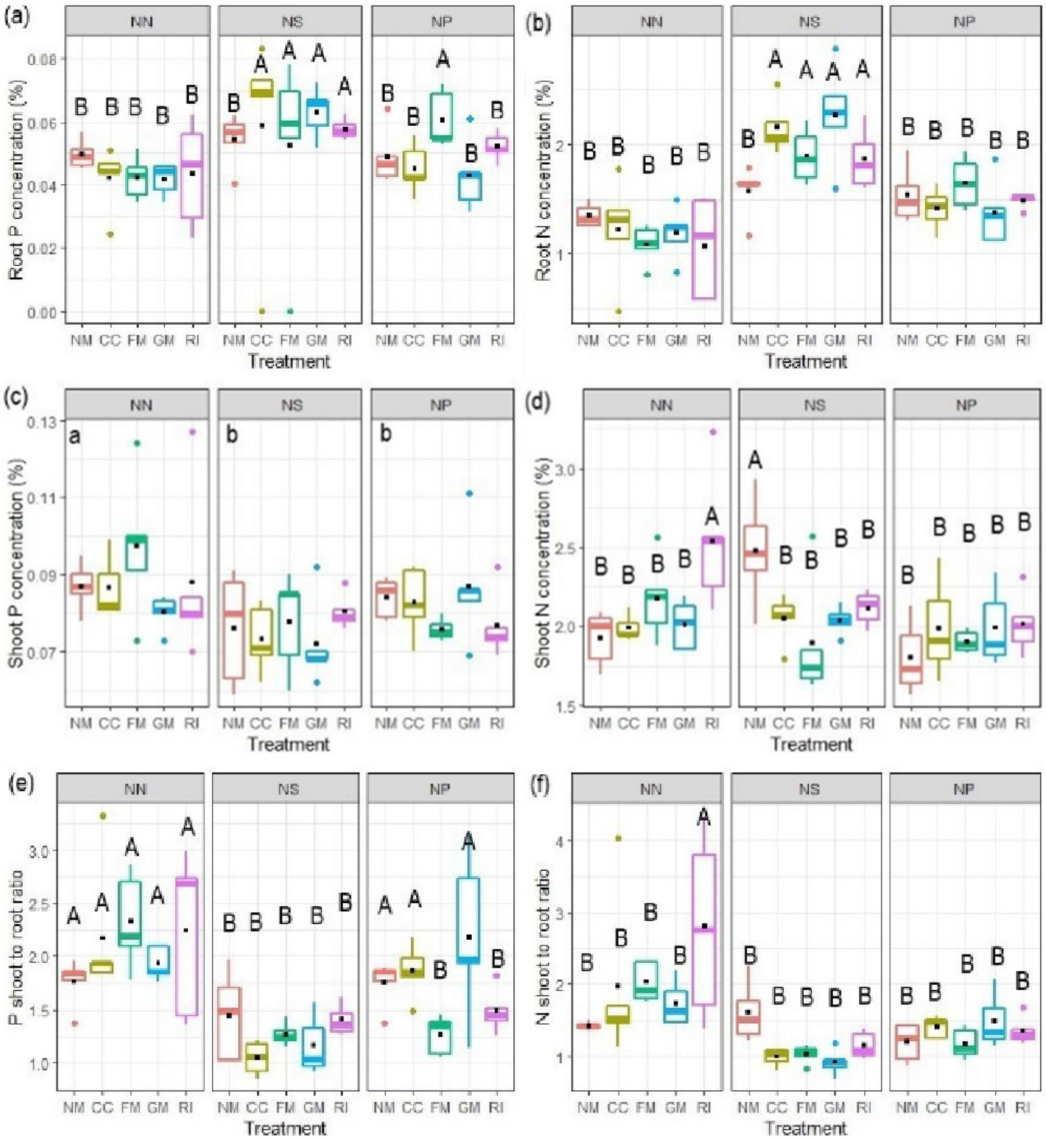
Concentrations of phosphorus (P) (**a**,** c**) and nitrogen (N) (**b**, **d**) in roots (**a**, **b**) and shoots (**c**, **d**), the shoot-to-root ratios of both elements (**e**, **f**) of tomato plants 60 days after inoculation with arbuscular mycorrhizal fungal (AMF) isolates, and subjected to different *M. incognita* treatments: no nematodes (NN), nematodes inoculated simultaneously with the AMF at planting (NS) or 2 weeks after AMF (NP). The AMF inoculations treatments were non-inoculated control (NM), inoculated with *C. claroideum* (CC), *F. mosseae* (FM), *Gi. margarita* (GM), or *R. intraradices* (RI). Different capital letters indicate significant differences across all treatments according to the DGC test (*p* < 0.05). Differences according to the DGC test (*p* < 0.05) for the nematode main factor effect are shown as lowercase letters at the upper left of each fungus panel when the interaction with nematode inoculation is not significant. In **f**, data were square root transformed to adjust for normality and homogeneity of variance. The box plot shows the 25% and 75% quartiles with median, 1.5 times interquartile range (as whiskers) and outliers. Outliers were included in all analyses. Black squares indicate the mean values of each treatment (*n* = 5). Statistics are presented in Table 2

Inoculation with AMF alone had no effect on P and N concentrations in roots, but significantly affected them in interaction with nematode treatments (Table 2; Fig. 3a, b). For both nutrients consistently, the interaction involved increased nutrient concentrations when AMF and nematodes were applied simultaneously. The reduction in root biomass was significantly correlated with an increase in root N concentration (*r*= −0.3, *p* = 0.01) but not with an increase in root P concentration ( *r*= −0.2, *p* = 0.09) (Fig. S1a, b).

In contrast to root P concentration, shoot P concentration was significantly reduced by both types of nematode application. AMF inoculation had no significant effect on this variable, neither alone nor in interaction with nematode application (Fig. 3c; Table 2). N concentration in shoots was significantly affected by nematode application and inoculation with AMF (Table 2). Inoculation with all AMF isolates reduced shoot N concentration when nematodes were applied simultaneously, but not in the other two nematode treatments (Fig. 3d). As in roots, shoot biomass was significantly negatively correlated with N concentration (*r*= −0.53, *p* = 0.0001), but not with P concentration (*r*= −0.005, *p* = 0.97) (Fig. S1c, d). The N to P ratio in shoots ranged from 21.5 to 26.5 (mean per treatment) and showed a significant interaction of AMF inoculation with nematode treatment (Table 2) that reflects the contrasting effects of AMF inoculation with and without nematode infection (Fig. S2a).

Inoculation with AMF had no overall effect on the shoot to root ratios of P and N, but significantly affected the ratios in interaction with nematode application (Fig. 3e, f; Table 2). In general, AMF inoculation tended to increase the P and N shoot to root ratios without nematodes, while an opposite tendency was observed with simultaneously inoculated nematodes.

As for the shoot dry weight, reduction of the shoot P content of non-mycorrhizal plants by nematode simultaneous infection was ameliorated by inoculation with all AMF (Fig. S2b). Shoot N content was significantly decreased by simultaneous infection with nematodes, while inoculation with AMF had no effect, neither alone nor in interaction (Fig. S2c, Table 2). Both parameters were correlated with shoot dry weight (P content: *r* = 0.81, *p* < 0.0001; N content: *r* = 0.75, *p* < 0.0001). P and N contents in roots were largely unaffected by the experimental factors, except for a significant effect of AMF on root P content, which was higher in FM than in the other inoculation treatments (Fig. S2d, e; Table 2).

### Nematode galling and reproduction

**Fig. 4:**
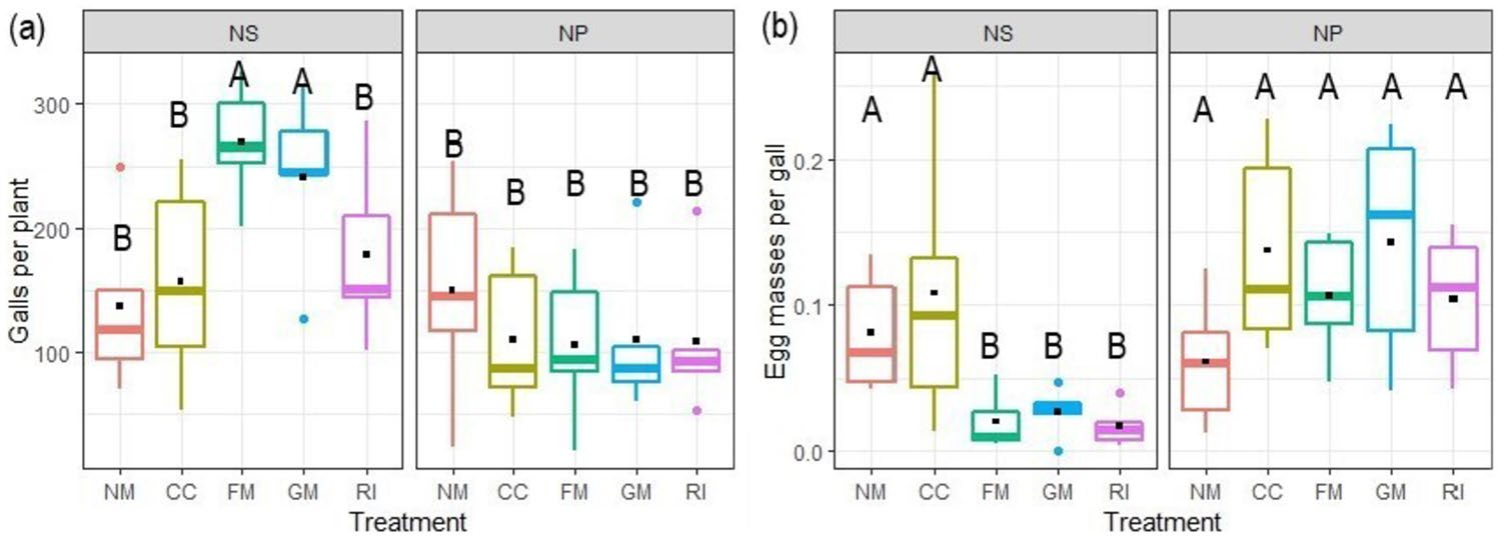
Total number of galls per plant (**a**) and egg masses per gall (**b**) produced by *M. incognita* in tomato plants infected with the nematode simultaneously with arbuscular mycorrhizal fungi (AMF) (NS), or 2 weeks later (NP). The AMF inoculations treatments were non-inoculated control (NM), inoculated with *C. claroideum* (CC), *F. mosseae* (FM), *Gi. margarita* (GM), or *R. intraradices* (RI). Different capital letters indicate significant differences across all treatments according to the DGC test (*p* < 0.05). The box plot shows the 25% and 75% quartiles, the median, the whiskers (1.5 times the interquartile range), and the outliers. Outliers were included in all analyses. Black squares indicate the mean values of each treatment (*n* = 5). Statistics are presented in Table 3

In NS, a higher number of galls was observed in plants inoculated with FM and GM than in the other AMF inoculation treatments (Table 3; Fig. 4a). This increase in the number of galls was not correlated with root dry weight (*r*=−0.03, *p* = 0.98). Egg masses per gall were lower with FM, GM, and RI, as compared to NM and CC in simultaneously infected plants, whereas no differences between the AMF treatments were detected after posterior nematode inoculation (Table 3; Fig. 4b). The reproduction factor of *M. incognita* was not affected by AMF treatment or time of nematode application (Table 3).

**Table 3:** Effect of inoculation with arbuscular mycorrhizal fungi (AMF), time of nematode application (nematode), and the interaction of both factors on *Meloidogyne incognita* parameters: galls per root system (galls), egg masses per gall (egg masses), and the reproduction factor (RF)

	**AMF**		**Nematode**		**AMF x Nematode**	
d.f.	4		1		4	
	F	*p*	F	*p*	F	*p*
Galls	1.15	0.34	15.46	**0.0002**	2.59	**0.05**
Egg masses	2.27	0.08	15.53	**0.0003**	2.59	**0.05**
RF	1.12	0.36	2.37	0.13	2.15	0.09

*F*-values and significances are given according to ANOVA (Type III Sums of Squares)

Significant effects are highlighted in bold

## Discussion

The main finding of this work is that all mycorrhizal isolates were able to ameliorate the drastic reduction in shoot biomass and P content caused by simultaneous infection with root-knot nematodes. This effect is consistent with some previous studies in which AMF were able to increase biomass in *Meloidogyne-*infected plants (Ahamad et al. 2023; Wang et al. 2023). In contrast, plants with posterior inoculation of nematodes were less affected in their growth than with simultaneous infection, even in non-mycorrhizal plants. This suggests that plants had more time to adapt to transplantation in the 2 weeks prior to nematode inoculation and therefore were less stressed by the infection. Similar results have been reported previously for a susceptible cultivar of alfalfa (*Medicago sativa*) (Grandison and Cooper 1986) and tomato (Talavera et al. 2001) infected with *Meloidogyne* spp., in which simultaneous nematode infection was more detrimental to the plants than posterior infection.

In the present study, AMF improved plant growth only when it was affected by simultaneous nematode infection. Similarly, Marro et al. (2014) reported that simultaneous AMF inoculation with the nematode *Nacobbus aberrans* could increase tomato growth, while no effect of AMF was observed with posterior nematode infection. In their case, however, the increase in biomass was associated with a reduction in nematode galls by AMF, contrary to our results. This variability observed in different studies reflects that the interaction between AMF and nematodes is multifactorial. For instance, the fertility of the system is one of the aspects that can cause differences between the results observed in the existing publications on the subject. It is therefore important to consider all the possible variables that can affect the interaction between nematodes and AMF in the rhizosphere and roots.

Unexpectedly, mycorrhizas did not increase the overall P concentration in tomato shoots in our experiment, even though the plants were P deficient, as indicated by their high shoot N to P ratio (Koerselman and Meuleman 1996). At the same time, the P content of the plant roots differed among experimental treatments, so it is unlikely that depletion of the entire P pool in the substrate caused this lack of mycorrhizal effects. Another possible explanation is the interplay between the direct and mycorrhizal pathways of P uptake (Smith et al. 2009; Smith and Smith 2012). The direct pathway absorbs P from the vicinity of the root epidermis and root hair cells, whereas the mycorrhizal pathway is activated by AMF root colonization and relies on specific Pi transporters that allow P to be translocated from the fungal intraradical mycelia to the root cortical cells (Smith and Smith 2011). In mycorrhizal plants, the direct pathway usually is downregulated, so that both pathways are complementary rather than additive in plant P uptake. In plants with an efficient direct pathway and low mycorrhizal responsiveness, this can lead to a lack of mycorrhizal effects on P uptake and growth even at high root colonization levels, despite a significant fungal contribution to plant P uptake, as shown specifically for tomato by Smith et al. (2004). However, as pointed out by Smith et al. (2009), the “hidden” P uptake via the mycorrhizal pathway may become “visible” when roots are damaged, and the capacity of the direct pathway is reduced by a root pathogen. This is consistent with the results of our experiment, where growth and P uptake of non-mycorrhizal plants were severely limited by nematodes, which was partially compensated by mycorrhiza.

Infection with nematodes increased P concentration in roots, which is in line with previous observations on different plants infected with *Meloidogyne* spp., such as tomato (Bergeson 1966; Carneiro et al. 2002; Oteifa and Elgindi 1962), pepper (Shafiee and Jenkins 1963), or *Vigna radiata* (Waghmare et al. 2022). *Meloidogyne* spp. release proteins from their pharyngeal glands to the roots and activate a cascade of immunological responses in plants, resulting in tissue lesions that form the feeding site and provide a continuous supply of nutrients to the nematode (Mitchum et al. 2013). This could explain the increase in root P and N concentrations associated with reduced growth. In addition, nematodes also reduced P translocation from roots to shoots, probably due to hypertrophy and hyperplasia of root xylem parenchyma cells due to the formation of feeding sites (Vilela et al. 2021), thus reducing P concentration in shoots. In the treatment with simultaneous nematode application, where the effect of nematodes on P partitioning was most pronounced and combined with reduced P content per plant, mycorrhiza further increased the P concentration in roots, but also P content in shoots. This suggests that AMF actually mitigated the negative effect of nematodes by supplying P to plants in a situation where direct P uptake by roots is limited. Although mycorrhizas did not increase the proportion of P translocated into the shoots, the increased supply to the roots helped to support the production of increased shoot biomass.

Similar effects as for P were found for N uptake and root-to-shoot translocation. However, some differences indicated a more important role for P than for N uptake in mycorrhizal mitigation of the damage caused by simultaneous nematode infection. The high N concentrations, as well as the highest N to P ratio in the shoots of non-mycorrhizal plants after simultaneous nematode infection, indicate that the nematode stress restricted their growth by aggravated P limitation. This interpretation is consistent with the absence of mycorrhizal effects on the total N uptake of plants simultaneously infected with nematodes. Also, the significant negative relationship of N concentration and biomass indicates growth limitation by another factor, presumably P. Plants can take up N via direct or mycorrhizal pathways, similarly to P, but contribution of the mycorrhizal pathway to the total N uptake of plants is unclear and probably less significant than in the case of P uptake (Smith and Smith 2011). Under N-deficient conditions, AMF even accumulate N in their mycelia and limit its uptake by the plant (Boussageon et al. 2022; Hodge and Fitter 2010; Ingraffia et al. 2020; Püschel et al. 2016; Treseder and Allen 2002). Therefore, it is not clear whether AMF could ameliorate N deficiency exacerbated by nematodes in a more N-limited system than in our experiment.

The described mycorrhizal effects generally were consistent among the four AMF isolates. This is remarkable in view of the significant differences in root colonization levels of the AMF isolates, because previous studies assumed that high AMF colonization should lead to elevated AMF-mediated biocontrol (e.g., Vierheilig et al. 2008). In the present work, *R. intraradices* and *F. mosseae* developed high total root colonization, while *Gi. margarita* and *C. claroideum* attained only low levels. This is in line with their root colonization characteristics as displayed in previous experiments (Blažková et al. 2021; Voříšková et al. 2017, 2019): rapid and extensive root colonization by *R. irregularis*, intermediate and variable root colonization ability by *F. mosseae* and *C. claroideum* isolates, and consistently low root colonization levels by *Gi. margarita*. Generally, *F. mosseae* and *R. intraradices* are considered to be rapid colonizers and highly infectious species, whereas *Gigaspora* spp. tend to produce extensive extraradical hyphae while their colonization of roots remains limited (Hart and Reader 2002; Powell et al. 2009). The similar level and mode of biotic stress alleviation despite different root colonization traits is consistent with the conclusion of Marro et al. (2022) that functional differences among AMF taxonomic groups may be smaller than previously thought.

The lack of effect of PPN infection on root colonization levels by any of the isolates contradicts previous suggestions that these nematodes may stimulate AMF colonization by altering the composition of root exudates to increase signals that act as a ‘cry for help’ (Rolfe et al. 2019). Conversely, competition for space and photosynthates has been hypothesized to reduce mycorrhizal colonization in the presence of *Meloidogyne* (da Silva Campos 2020; De Sá 2020). No effect of nematodes on AMF root colonization also has been reported (Anjos et al. 2010; Azcón-Aguilar and Barea 1996), suggesting that there is no general rule for this interaction, which may depend on the identity of AMF, PPN, and experimental conditions. Nevertheless, we found interesting effects of nematode infection on the formation of specific fungal structures: it significantly increased the frequency of vesicles in root colonization by *C. claroideum* and reduced the number of arbuscules by *F. mosseae* and *Gi. margarita.* Arbuscules are intracellular branched hyphal structures for nutrient exchange between the two symbionts (Tian et al. 2013), whereas vesicles are lipid reserves for fungal maintenance (Montero et al. 2019), formed only by some species. It has been suggested that the reduction of arbuscules and allocation of energy to fungal storage (vesicles) and reproductive structures (spores) is indicative for less mutualistic mycorrhizas (Buil et al. 2023; Cabello 1997; Johnson et al. 1997). Consequently, nematodes might have weakened the cooperation between the plant and its mycorrhizal symbionts, with AMF species responding differently to this stress. Interestingly, *R. irregularis* was the AMF species least responsive to nematode infection by altering its root colonization structures. Previously, this species was reported to be a generalist in terms of reliably supplying nutrients to its host plant under a range of nutrient conditions (Boussageon et al. 2022). Our results suggest that its interaction with the host plant also may be more robust to plant stress than that of the other AMF species tested in the experiment.

Surprisingly, the improved growth of mycorrhizal plants was not associated with a reduction in nematode development or reproduction. Similar results have been reported previously (Rodriguez-Heredia et al. 2020; Wang et al. 2023). Contrary to our expectations, more nematodes were able to form galls in the roots of *F. mosseae* and *Gi. margarita* than in control plants, indicating a higher level of nematode infection. The reason for this effect is unclear based on the available data. It is possible that this is related to the life cycles of these AMF isolates, such as a slower initial development of root colonization. However, this hypothesis needs to be examined.

Interestingly, we observed that not all galls contained egg masses at plant harvest, either because the eggs already hatched or they were not produced yet. However, the number of egg masses per gall was lower in *F. mosseae*,* Gi. margarita, *and *R. intraradices* than in non-mycorrhizal plants, suggesting that the nematodes were able to penetrate the roots, but a smaller proportion of them were able to reach the reproductive stage. In the case of the posterior nematode inoculation, even though the differences were non-significant, AMF plants tended to have fewer galls per plant, but more egg masses per gall than the non-mycorrhizal plants, which created an opposite pattern compared to simultaneous infection (see Fig. 4). This may suggest different mechanisms of the AMF protective effects depending on the timing of the nematode infection: simultaneous root colonization by both organisms may not reduce gall development although it affects the nematode ability to reproduce, while AMF root colonization prior to nematode infection may reduce nematode gall establishment.

## Conclusion

Our study demonstrated alleviation of nematode-induced stress by four AMF with different root colonization traits, which consistently improved plant P uptake and growth. Regarding the possible mechanisms of this effect, direct competition for space and carbon from roots (Schouteden et al. 2015) was not a likely cause in our study, as root penetration by nematodes was not significantly reduced in the presence of AMF, neither after simultaneous nor after posterior inoculation. Also, the number of galls was not related to the degree of root colonization by the different isolates.

Another mode of action of AMF against nematodes might be to increase plant tolerance to nematode attack. This was not exactly observed in our experiment as AMF did not improve the growth of non-infected plants per se. However, the alleviation of nematode-induced stress by AMF was clearly associated with improved P uptake. The fact that this stress alleviation was mediated by all AMF species, irrespective of the level of root colonization, highlights the importance of this mechanism. Considering the lack of plant benefits from AMF in the absence of nematodes, our results emphasize the need to focus on the interaction between stress and nutritional benefits of mycorrhizal fungi, which may be an important factor in stress alleviation. Indeed, it has been seen that P levels clearly affect plant defense responses to pathogens and herbivores (Chan et al. 2021). Mediation of stress resistance by the nutritional benefits of mycorrhizas also has been clearly demonstrated for other types of stress such as drought (Püschel et al. 2021), salinity (Qin et al. 2021), or low temperature (Chen et al. 2013). While non-nutritional benefits, such as induction of plant defenses by AMF, could be another explanation for our results, they were not explicitly addressed in our experiment. Further studies that focus on the variability among AMF species and specific pathways for the induction of resistance or tolerance will be important to ascertain how different AMF species affect the plant balance between growth and defense and to further elucidate the biological control potential of different AMF in soils.

### Supplementary information

Below is the link to the electronic supplementary material.

Supplementary materials (DOCX 573KB)
