# Supplementary material for: Divergent colonization traits, convergent benefits: different species of arbuscular mycorrhizal fungi alleviate Meloidogyne incognita damage in tomato
Source: Mycorrhiza. 2024 Mar 5;34(1-2):145–58. doi: 10.1007/s00572-024-01139-7 (PMC10998783; doi:10.1007/s00572-024-01139-7)
Supplement: Supplementary file 1 — Supplementary materials (DOCX 573KB) [file 572_2024_1139_MOESM1_ESM.docx]

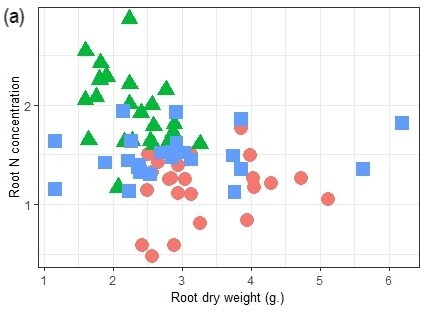

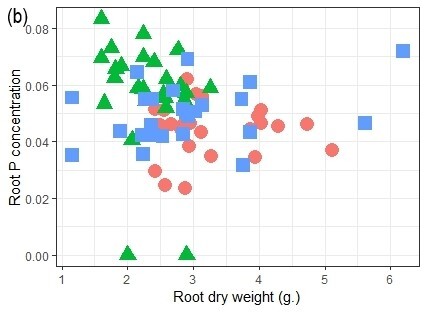

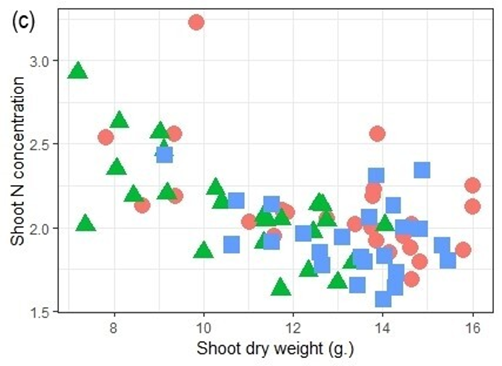

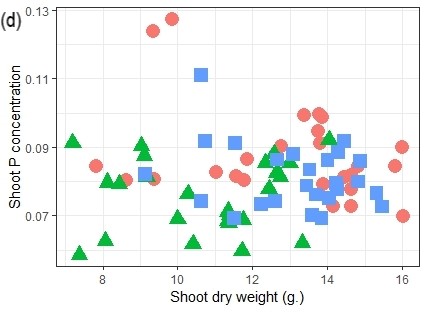


**Fig S1** Correlation of nitrogen (N) (a, c) and phosphorus (P) (b, d) concentrations in roots (a, b) and shoots (c, d) with root or shoot dry weights in tomato plants 60 days after inoculation with four arbuscular mycorrhizal fungi (AMF) isolates singularly, and subjected to different *M. incognita* treatments: no nematodes (circles), nematodes inoculated simultaneously with AMF (triangles), and nematodes inoculated two weeks after AMF (squares). All AMF treatments were included in the correlation (n=75 plants in total).

**
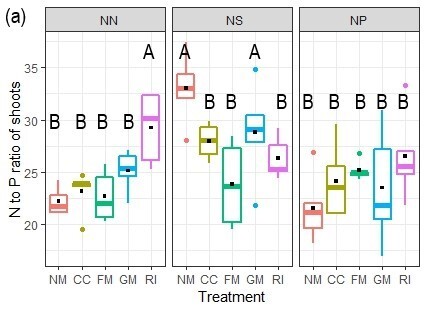

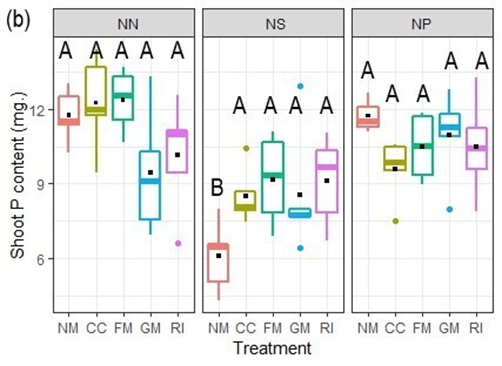
**

**
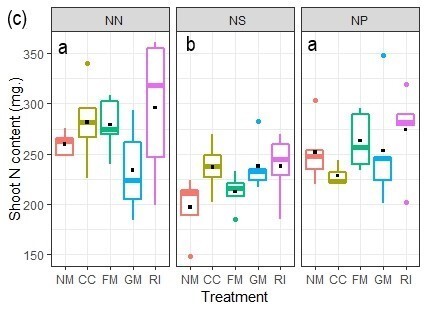

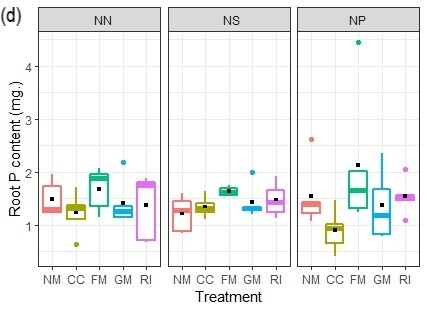
**

**
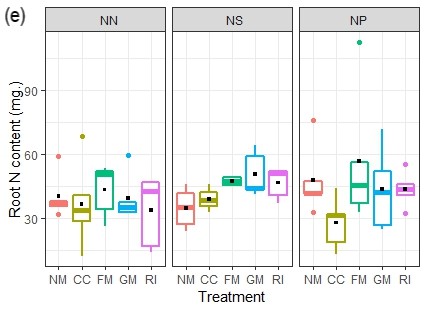
**

**Fig S2** Nitrogen (N) to phosphorus (P) ratio in shoots (a), P (b, d) and N (c, e) contents in shoots (b, c) and roots (d, e) of tomato plants 60 days after inoculation with four AMF isolates and subjected to different nematode treatments: no nematodes (NN), nematodes inoculated simultaneously with AMF at planting (NS) or 2 weeks after AMF (NP). The AMF inoculations treatments were: non-inoculated control (NM), inoculated with *C. claroideum* (CC), *F. mosseae* (FM), *Gi. margarita* (GM) or *R. intraradices* (RI). Different capital letters indicate significant differences across all treatments according to the DGC test (p<0.05). Differences according to the DGC test (p<0.05) for the AMF main factor effect are shown as lowercase letters at the upper left of each fungus panel when the interaction with nematode inoculation is not significant. Letters are not shown in (d) and (e) because neither the main factor effects nor their interaction were significant. Otherwise, when the interaction is not significant, differences among nematode treatments are represented as lowercase letters. The box plot shows the 25% and 75% quartiles with median, 1.5 times interquartile range (as whiskers) and outliers. Outliers were included in all analyses. Black squares indicate the mean values of each treatment (n = 5). Statistics are presented in Table 2
